# Single-issue advocacy in global health: Possibilities and perils

**DOI:** 10.1371/journal.pgph.0002368

**Published:** 2023-09-26

**Authors:** Katri Bertram, Madhukar Pai

**Affiliations:** 1 Partners of Impact, Berlin, Germany; 2 McGill School of Population and Global Health, McGill University, Montreal, Quebec, Canada; PLOS: Public Library of Science, UNITED STATES

*“There is no thing as a single-issue struggle because we do not live single-issue lives"—Audre Lorde*

In 2014, during the height of the West African Ebola crisis, Liberian nursing assistant Salome Karwah was profiled on the cover of Time Magazine as person of the year. Three years later, Time Magazine reported that Ms. Karwah had died in childbirth [1].

Ms. Karwah’s survival from Ebola, but tragic death from childbirth complications, is not a unique case. During the Covid-19 pandemic, many people who survived the virus died from other diseases and complications as health systems became strained or collapsed [2]. Every primary health care worker will have a story to share about a child surviving measles or malaria thanks to vaccines or timely emergency care, but suffering from or succumbing to malnutrition, violence, or diarrhea.

## A focused push for progress?

To address new or diverse health challenges, global health advocates play a big part in influencing global health practice. All of us are advocating for something or another, often because the issue is urgent, or because we really care about it.

For example, AIDS activism played a massive role in turning the tide on the HIV epidemic [3], and is held as a model for other areas [4]. Although AIDS activism focused on HIV, the movement went beyond treating the virus to addressing social determinants (e.g. criminalization of LGBTQ+ people, or gender-based violence) that drives the epidemic.

Advocacy also played a key role in the eradication of smallpox, and in greatly reducing the burden of polio. During the Covid-19 pandemic, many advocated for Covid vaccine equity because billions of lives were at risk, and we needed to vaccinate the world [5].

There are many reasons why single-issue advocacy is popular. It is often easier to rally people around a clear, simple call to action. Donors often push for it, and everyone needs to see quick wins and success stories to keep motivated.

But single-issue advocacy comes with some dangers. First, when we advocate for single issues, we sometimes lose sight of the broader context that erodes progress in our area. For example, even when Covid-19 vaccines were more easily available, their uptake was blunted by weak health systems, health workforce shortages, vaccine hesitancy, broken supply chains, and competing needs [5].

Second, when we are laser-beam focused on a single issue, we can end up competing instead of collaborating. For example, we end up pitting one area against another (e.g. HIV funding matters more than funding for TB or malaria) because we think our specific issue or cause is more important than others. We see this reflected in how people advocate and frame their campaigns: “disease X kills more people than TB, AIDS and malaria combined.”

Paul Farmer had cautioned about this scarcity mindset which pits condition A against B. He encouraged us to ‘counter failures of imagination’ and demand more resources (i.e. to grow the pie), instead of competing amongst ourselves for fixed resources [6].

Third, single issue advocacy ignores people’s realities. As Audre Lorde put it, “we do not live single-issue lives [7].” All of us have intersectional identities and multiple health needs. In fact, multi-morbidity is common [8], especially for vulnerable people such as those in conflict settings or living in poverty, but also for all of us as we grow older.

For example, people with TB are often co-infected with HIV, and tend to be malnourished or have diabetes. So, what happens when someone with TB needs nutritional support or anti-retrovirals (ARVs) or mental health services? Will advocating for TB alone solve those issues? Imagine curing TB but not being able to provide them ARVs for HIV, or insulin for diabetes?

Fourth, single-issue advocacy imposes a tunnel vision, and that comes with the inability to expand the scope for solutions. We push for capacity and funding for narrow silver-bullet solutions, and believe that things will be fine, if only we can fix this or that single problem in a complex, inter-connected world [9].

Frequently, we find that fixing one issue merely shifts the problem to another part of the system. For example, advocacy and projects to scale-up malaria rapid testing did reduce unnecessary use of anti-malarial medicines but focusing only on a single disease simply led to overuse of broad-spectrum antibiotics [10].

Climate justice activists warn us about the dangers of being tunnel visioned and only focus on carbon emissions, without addressing the social, economic and political structures that have put our futures at risk [11]. “Trapped by tunnel vision, the chance to imagine radical solutions for sustainability is stolen from the collective imagination by those benefiting from the current economic system,” argue Deivanayagam and Osborne [11]. Single-issue advocacy tends to limit or narrow the potential solutions we can imagine.

## How do we deal these perils of single-issue advocacy?

There are no easy solutions, but we can advocate for universal health coverage (UHC), stronger health systems, and greater investments in issues that affect health outcomes overall, in addition to advocating for whatever we are most passionate about.

UHC is the one thing that grows the pie and unites us all, regardless of which area or population group we care about. There is no area in global health that will not benefit from UHC and a stronger, more equitable health system. The primary principle of UHC is universality—that all people are covered. Advocacy for UHC is people-centered, not focused on specific population groups or health interventions [12]. While every disease or interest group is quick to emphasize that their area is critical for UHC, they also need to shift their single-issue advocacy to cover UHC and stronger health systems. In short, everyone in health must become an advocate for health as a human right and UHC.

For example, those who advocate for global surgery are aware that surgery cannot be performed safely without a decent health infrastructure that includes anesthesia and post-operative support–they need to advocate for UHC. Tuberculosis will greatly benefit from a stronger primary care system since most people with TB first seek care at the level of primary care. Diagnostics for cancer or diabetes will improve when countries adopt a broader essential diagnostics list and include them in national UHC benefits packages.

To improve health outcomes for all people, we need to understand the importance of bigger systemic changes and other issues that impact our focus area. We need to read beyond our narrow areas, meet with people with diverse expertise and experiences, and learn how to shape a broader agenda. Even UHC, at some level, is constrained within the health space, when some of biggest challenges for health lie in the social, economic and political realm. Widening economic inequities, Global North’s domination of geopolitics, racial capitalism, racism, and heteropatriarchy are massive issues that UHC cannot directly address [13].

Global health is full of false dichotomies [14]. We must not create yet another false dichotomy between single-issue vs broader advocacy. We need to use both approaches and smartly. This requires changes to how we communicate, fundraise, and advocate in global health [15]. A better understanding of the possibilities and perils of both approaches could help all of us navigate this dilemma we all face daily.
